# Association of Vitamin D Supplementation with VDR and CAMP Expression and Ulcer Healing in Diabetic Foot Infection Involving Biofilm-Forming Bacteria: An Exploratory Prospective Cohort Study

**DOI:** 10.3390/ph19101508

**Published:** 2026-09-23

**Authors:** Ruby Benson, Saleena Ummer Velladath, Seetharam Shiva Prasad, Sudheesh Nittur, Barnini Banerjee, Gabriel Sunil Rodrigues, Mahadev Rao, Raghu Chandrashekar Hariharapura, Murali Munisamy, Shashidhar Vishwanath, Chiranjay Mukhopadhyay, Varun Kumar Sudha Gururaj, Sonal Sekhar Miraj

**Affiliations:** 1 Department of Pharmacy Practice, Manipal College of Pharmaceutical Sciences, Manipal Academy of Higher Education, Manipal 576104, India; ruby.benson@learner.manipal.edu (R.B.); mahadev.rao@manipal.edu (M.R.); 2 Department of Medical Laboratory Technology, Manipal College of Health Professions, Manipal Academy of Higher Education, Manipal 576104, India; saleenaummer@nu.edu.om; 3 Department of General Surgery, Kasturba Medical College, Manipal Academy of Higher Education, Manipal 576104, India; prasad.ss@manipal.edu (S.S.P.); gabyrodricks@gmail.com (G.S.R.); 4 Manipal Institute of Virology, Manipal Academy of Higher Education, Manipal 576104, India; sudheesh.n@manipal.edu; 5 Department of Microbiology, Kasturba Medical College, Manipal Academy of Higher Education, Manipal 576104, India; barnini.banerjee@manipal.edu (B.B.); shashidhar.v@manipal.edu (S.V.); chiranjay.m@manipal.edu (C.M.); 6 Department of Pharmaceutical Biotechnology, Manipal College of Pharmaceutical Sciences, Manipal Academy of Higher Education, Manipal 576104, India; raghu.c@manipal.edu; 7 Department of Translational Medicine, All India Institute of Medical Sciences, Bhopal 462020, India; murali.tmc@aiimsbhopal.edu.in; 8 Department of Applied Statistics and Data Science, Prasanna School of Public Health, Manipal Academy of Higher Education, Manipal 576104, India; varunsg.kumar@manipal.edu

**Keywords:** diabetic foot infection, vitamin D, vitamin D receptor, CAMP, LL-37, biofilm, wound healing

## Abstract

**Background/Objectives:** Diabetic foot infections (DFIs) are a major cause of morbidity and lower-limb amputation. Vitamin D exerts immunomodulatory effects through vitamin D receptor (VDR), which regulates the cathelicidin antimicrobial peptide (CAMP) gene that encodes cathelicidin (LL-37). However, their association with DFIs remains poorly understood. This study investigated the association between vitamin D supplementation, the vitamin D–VDR–CAMP–LL-37 pathway, and ulcer healing in DFIs involving biofilm-forming bacteria. **Methods:** This study (September 2022–March 2026) included 16 DFI patients in the study group (SG), 16 in the reference group (RG), and 16 healthy controls (HCs). The SG received vitamin D supplementation (60,000 IU) in addition to standard care, whereas the RG received standard care alone. Participants were followed up for 4 months. Vitamin D assessment (electrochemiluminescence immunoassay), VDR and LL-37 quantification (ELISA), biofilm formation (tissue culture plate method), and VDR and CAMP gene expression (mRNA quantitation) were all considered. **Results:** At baseline, serum vitamin D, VDR protein, and LL-37 levels differed significantly among the groups (*p* ≤ 0.002), whereas VDR and CAMP gene expressions did not. Following supplementation, the SG showed significant increases in serum vitamin D, VDR protein and LL-37 (*p* ≤ 0.001). At the four-month follow-up, there was no significant difference in VDR and CAMP gene expression or in ulcer healing between the groups. Although a higher proportion of participants in the SG achieved ulcer healing, the difference was not statistically significant. **Conclusions:** Although changes in the circulating elements of the vitamin D-VDR-LL-37 pathway were noted with physician-prescribed vitamin D supplementation, no statistically significant association with ulcer healing was observed. This finding requires validation through a thorough investigation.

## 1. Introduction

The International Diabetes Federation states that approximately 590 million individuals aged 20–79 years suffer from diabetes mellitus (DM) worldwide [1]. Globally, 1 in 7 adults has DM in India [2]. DM is a metabolic disorder that is characterized by hyperglycaemia. It is increasingly common because of several factors, including urbanization, sedentary lifestyles, obesity, and an aging population [3]. Chronic hyperglycaemia causes DM complications, such as diabetic foot infections (DFIs), which cause significant disability [4]. The lifetime risk of developing a diabetic foot ulcer (DFU) is approximately 25% among people with DM worldwide. Among them, 70% require prolonged hospital stays and often require multiple surgeries, including debridement and revascularization [5]. If not treated in time and effectively, infection can progress to deep tissue destruction, osteomyelitis, and gangrene, resulting in lower-limb amputation [5]. As reported, chronically infected patients often have low vitamin D levels, along with modifications in vitamin D receptor (VDR) signalling and metabolism [6]. Furthermore, the mortality risk in diagnosed DFI is approximately three times greater than in patients without DFI [6]. A 2022 study found that managing DFIs in India resulted in annual out-of-pocket expenses of approximately ₹81,000, placing a significant financial strain on affected households [7].

Biofilm-forming bacteria complicate the treatment of DFI wounds [8]. Studies have shown that microbial biofilms formed by multiple organisms in the wounds of patients with DFI delay wound healing and worsen the prognosis [9,10]. These biofilms inhibit antibiotic penetration into the wound bed, thereby promoting the emergence of multidrug-resistant bacteria.

Vitamin D is increasingly recognized for its traditional role in immunomodulation [11]. Active vitamin D, whether circulating or locally generated, is involved in the VDR-mediated regulation of the cathelicidin antimicrobial peptide (CAMP) gene [11]. This gene encodes the inactive precursor protein hCAP18, which is subsequently cleaved to produce the active peptide, LL-37 [11,12]. In peripheral blood, neutrophils express CAMP mRNA and synthesize and store hCAP18 in their granules [13]. Proteinase 3 cleaves hCAP18 upon release, forming LL-37, an antimicrobial peptide (AMP) [13]. In the skin, CAMP is predominantly expressed by resident epidermal keratinocytes. Its expression is upregulated in response to injury, infection, and inflammation [11,12,13].

LL-37 exhibits broad antimicrobial activity against both Gram-negative and Gram-positive bacteria, primarily by disrupting bacterial membranes [13,14,15,16]. It also has antiviral activity, particularly against enveloped viruses [13,14,15] and plays a role in innate host defense. In addition, it may be involved in wound repair because of its role in angiogenesis [14]. Given the impaired host defense and delayed wound healing associated with DFIs, changes in the vitamin D–VDR–CAMP/LL-37 pathway may have clinical relevance. However, the effect of vitamin D supplementation on this pathway and its potential relationship with wound-healing outcomes in DFI involving biofilm-forming bacteria remains to be explored.

Despite accumulating evidence supporting the immunomodulatory role of vitamin D, few studies have investigated its molecular effects in patients with DFI [17,18,19]. Most previous studies have focused on the association of serum vitamin D with clinical outcomes in DFI [18,19,20]. In contrast, few studies have investigated downstream changes in VDR expression and the activation of antimicrobial genes, such as CAMP, in other diseases, including leprosy and COVID-19 [21,22]. The expression of the VDR gene was studied in vitro in healthy subjects and patients with non-infected DFUs [23]. The expression of the VDR gene did not change significantly. However, to our knowledge, no study has examined VDR gene expression in DFI. Specifically, it is still unknown how vitamin D supplementation, VDR gene expression, LL-37 production, and ulcer healing are related. This knowledge gap is particularly important in the context of infections involving organisms that form biofilms, which are highly resistant to treatment. By understanding these molecular reactions, vitamin D supplementation could enhance wound healing and infection management alongside standard care. The study compared VDR and CAMP gene expression and related biomarkers among patients with DFIs (study group [SG] and reference group [RG] and healthy controls [HCs]). We further investigated the association of vitamin D supplementation with VDR and CAMP gene expression, serum VDR and LL-37 levels, and wound healing in DFUs infected with biofilm-forming bacteria.

## 2. Results

### 2.1. Comparison of Characteristics of the Study Population at Baseline

Significant age differences were observed among the three groups (*p* < 0.001). The HCs had the lowest median age of 32.5 (9.25) years, followed by SG at 52.5 (10) years and RG at 61.5 (7.5) years. Serum vitamin D levels differed significantly between the groups (*p* = 0.001). The HCs had the highest median vitamin D level at 21.9 (17.4) ng/mL, followed by RG at 16.3 (5.2) ng/mL and SG at 11.4 (7.15) ng/mL. The HC group had a median serum VDR concentration of 1.14 (0.39) ng/mL, compared with 0.79 (0.41) and 0.76 (0.34) in the RG and SG, respectively (*p* = 0.002). Serum LL-37 levels also differed (*p* = 0.001), with HCs having the highest median concentrations at 1.89 (1.08) ng/mL, followed by RG at 1.26 (0.79) and SG at 1.03 (0.42). The relative expression of the VDR gene did not differ significantly between the groups (*p* = 0.059). Similarly, the CAMP gene expression did not differ significantly between the groups (*p* = 0.241) (Table 1).

### 2.2. Comparison of Characteristics of the Study Populations at Four-Month Follow-Up

At the four-month follow-up, serum vitamin D, VDR protein, and LL-37 levels, along with peripheral-blood VDR and CAMP gene expression, were compared among the SG (*n* = 11), RG (*n* = 16), and HCs (*n* = 16). Significant differences were observed among the three groups in terms of serum vitamin D levels (*p* < 0.001). The SG had significantly higher serum vitamin D levels than the RG, with levels comparable to those of the HC participants (21.3 vs. 11.1 vs. 21.9 ng/mL, respectively). Furthermore, significant differences in serum VDR concentrations were observed among the three groups (*p* = 0.001). Similarly, serum LL-37 levels differed significantly among the three groups (*p* = 0.001). The SG exhibited significantly higher LL-37 levels than the RG (1.89 vs. 0.60 ng/mL). In contrast, the RG had significantly lower LL-37 levels than the HCs (0.60 vs. 1.89 ng/mL). The expression of the VDR gene did not differ significantly among the three groups (*p* = 0.067). However, pairwise analysis revealed significantly higher VDR gene expression in the SG than in the RG (*p* = 0.032). CAMP gene expression was not significantly different between the groups (*p* = 0.346), and post-hoc analysis revealed no pairwise differences (Table 2).

### 2.3. Comparison of Wound Outcome Between RG and SG at Four-Month Follow-Up

During the four-month follow-up, healing of DFUs was observed in 3 (18.8%) patients in the RG and 4 (36.4%) in the SG, while nonhealing was documented in 13 (81.2%) and 7 (63.6%) patients, respectively. Although the ulcer healing rate was higher in the SG than in the RG, a statistically non-significant difference (*p* = 0.391) was observed, as shown in Figure 1.

### 2.4. Association Between Vitamin D Status and Bacterial Isolates in the SG at Four-Month Follow-Up

Biofilm-forming bacterial isolates, including methicillin-sensitive *Staphylococcus aureus*, methicillin-resistant *Staphylococcus aureus*, *Proteus mirabilis*, *Pseudomonas aeruginosa*, and *Klebsiella pneumoniae*, were more frequently observed among participants with vitamin D deficiency (*n* = 8, 66.7%). Lower frequencies were observed among those with vitamin D insufficiency (*n* = 3, 25.0%) and those with sufficient vitamin D status (*n* = 1, 8.3%). Conversely, non-biofilm-forming bacterial isolates such as *Citrobacter freundii*, *Providencia stuartii*, and *Streptococcus pyogenes* were more frequently observed in participants with sufficient vitamin D levels (*n* = 2, 50.0%). Lower frequencies were observed among those with deficient (*n* = 1, 25.0%) or insufficient (*n* = 1, 25.0%) vitamin D status. However, the differences were not statistically significant (*p* = 0.163). This finding indicates that the vitamin D status of SG participants was not significantly associated with the presence of biofilm-forming or non-biofilm-forming bacterial isolates in their wounds (Table 3).

### 2.5. Association Between Vitamin D Status and Ulcer Healing Outcomes in the SG at Four-Month Follow-Up

Ulcer healing was more frequently observed among participants with sufficient vitamin D status (*n* = 2, 50.0%) than among those with a deficient (*n* = 1, 25.0%) or insufficient vitamin D status (*n* = 1, 25.0%). In contrast, non-healing ulcers were noted more frequently in vitamin D-deficient patients (*n* = 4, 57.1%) in the SG compared with those with insufficient (*n* = 2, 28.6%) or sufficient (*n* = 1, 14.3%) vitamin D levels. Nevertheless, the study found no statistically significant association between vitamin D status and ulcer healing outcomes in the SG group (*p* = 0.727) (Table 4).

### 2.6. Correlation Between Vitamin D, Biomarkers, and Expression Levels of the VDR and CAMP Genes in SG Patients at Four-Month Follow-Up

At four-month follow-up, Spearman’s rank correlation analysis in the SG revealed significant positive correlations between serum vitamin D concentration and VDR protein levels (r = 0.682, *p* = 0.025) and LL-37 levels (r = 0.825, *p* = 0.002). Significant positive correlations were also observed between serum vitamin D concentration and VDR (r = 0.618, *p* = 0.048) and CAMP (r = 0.655, *p* = 0.034) gene expression (Table 5).

### 2.7. Comparison of Baseline Characteristics of Four-Month Follow-Up Participants and Those Lost to Follow-Up

Of the 16 SG participants, 11 completed the four-month follow-up, and 5 were lost to follow-up. The baseline characteristics were similar for participants who completed follow-up and those who did not (all *p* > 0.05) (Table 6).

## 3. Discussion

The current study assessed variations in vitamin D, VDR protein, and LL-37 levels in DFI patients and HCs, as well as VDR and CAMP expression. Additionally, it examined how physician-prescribed vitamin D supplementation affects the clinical outcomes and molecular biomarkers of DFI patients.

The significant age disparity among the three groups corresponds with the natural progression of T2DM and DFI, which tend to be more common in older adults. This prevalence is attributed to prolonged exposure to blood sugar spikes and the cumulative effects of weakened immunity, peripheral neuropathy, and other factors [24]. Due to decreased angiogenesis, decreased collagen synthesis, and impaired inflammatory regulation, advanced age is an independent predictor of DFU and prolonged wound healing [25,26]. Older individuals often exhibit a greater prevalence of comorbidities that negatively impact tissue repair [27]. The HC group, devoid of diabetes or related complications, was anticipated to be younger than the other groups [28].

Significant differences in serum vitamin D concentrations between the three groups are consistent with mounting evidence that vitamin D deficiency is common in DFI patients. DFI is linked to vitamin D deficiency in previous studies [18,29,30,31]. However, some studies provide insufficient evidence linking vitamin D levels to DFI. The variation in testing methods may account for these discrepancies [20,32]. Our study utilized an electrochemiluminescence immunoassay to measure vitamin D levels, whereas other studies employed ELISA [20,32]. Serum vitamin D was lower in DFI patients (SG and RG at baseline) than in HCs. Another study found no significant difference in serum vitamin D levels between DM patients with and without foot infections [17]. However, Vitamin D deficiency worsened the prognosis of DFIs [19].

The results of a prior clinical study were in line with the observed drop in VDR protein levels in DFI [33]. According to this study, DFI patients had considerably lower serum levels of VDR vitamin D. Furthermore, reduced VDR levels were independently associated with ulcer development, suggesting a potential role for VDR signalling in DFI pathogenesis [33]. Similar findings were observed in a study of 169 patients with DFI in the same setting [29]. Together, these findings indicate that diminished VDR availability may hinder antimicrobial responses and wound healing in patients with DFI.

The present study revealed variations in serum LL-37 concentrations among the three groups. The lower LL-37 levels observed might be due to vitamin D deficiency and disruption of VDR signalling. An in vitro study showed that treatment with 1,25-dihydroxyvitamin D3 significantly enhanced LL-37 production in keratinocytes from DFUs and stimulated wound-healing responses [23]. LL-37 regulates inflammation, tissue regeneration, and infections [34]. Collectively, these findings suggest that restoring vitamin D-VDR signalling can boost LL-37 production and promote wound repair.

VDR gene expression did not differ among HCs, RG, and SG participants (*p* = 0.059). In addition, no significant difference in CAMP gene expression between the groups was observed (*p* = 0.241). The near-significant reduction in VDR gene expression is biologically plausible because chronic hyperglycaemia, oxidative stress, and inflammation are known to suppress vitamin D signalling [35]. A review explained that vitamin D-mediated activation of VDR is essential for the transcription of antimicrobial peptides (AMPs) that enhance epithelial defense and tissue repair [22]. However, gene expression measured in peripheral blood may not accurately reflect the local transcriptional activity in wound tissues, where these pathways are most active [36]. Previous studies have demonstrated the tissue-specific expression of VDR, CAMP, and LL-37 in DFU biopsies and wound-margin tissues [23,25,37]. Therefore, the present study could not determine the local or cell-specific expression. Hence, the present findings should be interpreted as associations between systemic and peripheral blood, rather than as direct evidence of in situ VDR–CAMP/LL-37 activity. Future studies assessing both blood and wound tissue samples from the same patients are needed to clarify the relationship between systemic and local VDR–CAMP/LL-37 activities. Wound tissue analysis and longitudinal vitamin D supplementation studies are required to understand the transcriptional dynamics of these pathways.

At the four-month follow-up, the SG had significantly higher serum vitamin D levels than the RG, which were comparable to those in the HCs. This suggests that vitamin D levels in the SG at follow-up were restored to levels comparable to those of the HCs group. Similarly, Gao et al. found that vitamin D-supplemented DFI patients showed reduced infection rates and accelerated wound healing [37].

Additionally, serum VDR concentrations differed significantly between the three groups. However, pairwise comparisons showed that VDR concentrations in the SG at four-month follow-up were not significantly different from those in the RG at four-month follow-up or in HCs. Therefore, the VDR protein showed a trend toward improvement in the SG. Similarly, the SG participants had significantly higher LL-37 levels than the RG but not the HCs, indicating that their levels had returned to HC levels. These findings indicate that vitamin D supplementation was associated with changes in LL-37 concentrations [15,16].

Pairwise analysis revealed that the SG participants had significantly higher VDR expression than the RG participants, but there was no statistically significant difference in VDR expression among the three groups. Vitamin D supplementation may boost VDR gene expression, thereby supporting VDR-mediated signalling pathways [15,37]. Vitamin D supplementation may partially restore VDR gene expression to physiological levels, as the SG did not differ significantly from the HCs. However, chronic inflammation and DFI may reduce VDR expression in RG by impairing vitamin D signalling [17]. Vitamin D supplementation may affect VDR transcription in patients with DFI, but more research is needed to confirm this.

Vitamin D supplementation did not alter SG CAMP gene expression but increased serum LL-37 levels to levels observed in HCs. CAMP gene expression was measured in peripheral blood; however, neutrophils, macrophages, and keratinocytes produce LL-37 at the wound site [38]. Thus, vitamin D supplementation may have increased local LL-37 production and innate immune function without increasing peripheral blood CAMP gene expression. Hence, the observed increase in LL-37 should not be interpreted as evidence of increased CAMP transcription.

Among patients who achieved wound healing, the proportion in the SG was higher than that in the RG, who received standard care alone. The difference was not statistically significant, and no clear association was identified between the vitamin D supplementation status and wound healing.

Furthermore, bacteria isolated from wound samples of vitamin D-deficient SG participants exhibited greater biofilm-forming capacity than those isolated from participants with insufficient or sufficient vitamin D levels. However, this difference was not statistically significant (*p* = 0.163).

A greater percentage of SG participants with sufficient vitamin D levels achieved wound healing compared with those with deficient or insufficient levels. Nevertheless, no statistically significant association was identified between vitamin D levels and ulcer healing rates. Vitamin D is known to promote wound repair through mechanisms such as immunomodulation, enhancement of antimicrobial peptide production, regulation of inflammation, and stimulation of keratinocyte proliferation and angiogenesis [9,12]. Moreover, ulcer healing is a complex process influenced by factors such as infection severity, glycemic control, vascular status, neuropathy, and wound care [39]. Therefore, larger prospective studies are needed to determine whether adequate vitamin D status independently contributes to improved healing outcomes in DFUs.

In the SG, serum vitamin D concentration was positively correlated with serum VDR protein, LL-37 levels, and VDR and CAMP gene expression. These measurements provide information on systemic protein levels and circulating gene expression patterns. However, it cannot establish the corresponding protein concentrations or transcriptional activity in wound tissue.

Furthermore, no significant differences in baseline characteristics were found between the SG participants who completed the four-month follow-up and those who were lost to follow-up. The small number of participants, especially those lost to follow-up, limited the ability to detect baseline differences. Selection bias due to attrition cannot be ruled out; therefore, follow-up results should be interpreted cautiously.

Several limitations should be considered when interpreting the findings of this study. First, debrided wound tissue was not collected for gene expression or protein analysis. Therefore, these circulating measurements may not reflect transcription or protein activity in the wound microenvironment. VDR and LL-37 protein concentrations were measured in the serum, whereas VDR and CAMP mRNA expression were assessed in the peripheral blood. These measurements provide minimally invasive and clinically feasible indications of systemic protein levels and circulating gene expression patterns. However, these may not directly reflect VDR and CAMP transcription or VDR and LL-37 protein concentrations within the local wound microenvironment. Previous studies have demonstrated that LL-37 expression differs between healing wounds and chronic ulcer tissues [23,25]. These findings further support the importance of direct assessment at the wound site. Therefore, the present findings should be interpreted as associations between systemic and peripheral blood, rather than as direct evidence of in situ VDR–CAMP/LL-37 activity. Future studies assessing both blood and wound tissue samples from the same patients are needed to clarify the relationship between systemic and local VDR–CAMP/LL-37 activities. Loss to follow-up was another limitation of this study, as many patients were referred from distant districts to our tertiary referral centre for severe DFIs. Some patients missed their scheduled four-month follow-up appointment, possibly due to difficulties related to distance and travel after initial management, which limited the ability to conduct a complete longitudinal assessment of treatment outcomes. Another limitation is the significant age discrepancy between the HC group and the DFI patient groups. The median age of the HCs was considerably lower (32.5 years) than that of the SG (52.5 years) and the RG (62.5 years). This age variation could potentially influence immune function and the biomarkers assessed in this study. Such a disparity may be a confounding factor to consider when interpreting the findings. A limitation of the sample analysis is that some of the ELISA measurements, especially two VDR and two LL-37 measurements, were near the lower limit of the ELISA assay range (0.63 ng/mL and 1.56 ng/mL, respectively). This may have limited the quantitative precision of these results; therefore, they should be interpreted with caution.

Furthermore, ulcer healing was determined using routine clinical documentation rather than standard serial wound measurements or calibrated photographs. The ulcers recorded as healed had no follow-up measurements; therefore, changes in wound area and the percentage reduction could not be calculated. Therefore, the healing results must be considered exploratory based on clinical records.

The exclusion of patients with peripheral vascular disease in this study reduced its representativeness and external validity. To determine whether their associations are present in the broader DFI population, future studies should include patients with varying degrees of vascular impairment.

Furthermore, the increase in serum vitamin D levels among the SG participants was consistent with the exposure to supplementation. However, individual adherence could not be confirmed because compliance was not objectively assessed in this study. Therefore, supplementation-related findings should be interpreted cautiously.

Some *p*-values were close to the conventional significance level (*p* < 0.05). Owing to the exploratory design and small sample size, these results may be sensitive to sampling variability and are not conclusive. More extensive prospective studies are required to confirm these associations and their potential implications for DFI wound healing.

## 4. Materials and Methods

### 4.1. Study Details

An exploratory prospective cohort study was conducted with 48 participants. The study included 16 patients with DFI in the SG, 16 patients with DFI in the RG, and 16 HCs. Patients with DFI were assigned to either the SG or RG based on their vitamin D supplementation status in the General Surgery Department at Kasturba Hospital (KH), Manipal, India, between September 2024 and March 2026. Vitamin D supplementation was prescribed by the treating physician as part of routine clinical care. Patients in the RG received only standard of care, whereas those in the SG received 60,000 IU of vitamin D once weekly for 4 months in addition to standard of care. All patients with DFI were monitored for four months to assess clinical and biochemical outcomes. However, adherence to these supplements was not systematically assessed. The Institutional Ethics Committee (IEC) of Kasturba Medical College and Kasturba Hospital (KMC & KH), Manipal, approved the study under the reference number IEC: 676/2020. The study followed the Declaration of Helsinki 2013.

### 4.2. Enrolment of Study Subjects

#### 4.2.1. Inclusion and Exclusion Criteria

DFI patients: Diabetic patients aged ≥18 years, regardless of sex, diagnosed with DFI according to the IWGDF/IDSA infection grading system were included. Exclusion criteria included hepatic or renal disorders that could affect vitamin D metabolism. Patients with conditions that impair vitamin D absorption, individuals with peripheral vascular disease, and patients who received immunosuppressants, chemotherapeutic agents, or corticosteroids within the previous 30 days were excluded. Pregnant and lactating women were also excluded from the study. Patients who did not consent to participate were excluded from the study.

HCs: Males and females aged ≥18 years without diabetes or other comorbidities. Patients with diabetes, hypercalcemia, skin cancer, or those receiving steroids, multivitamins, or immunosuppressants were excluded from the study. Women who were pregnant or breastfeeding were also excluded from the study.

#### 4.2.2. Recruitment of DFI Patients and HCs

A total of 252 DFI patients were screened for eligibility. Among them, 72 had peripheral vascular disease, 52 had wounds with negligible bacterial growth on culture, and 18 had renal disease. Additionally, 9 had osteomyelitis, 7 had hepatic disorders, and 5 declined to provide consent. Consequently, 89 patients met the eligibility criteria. Three patients were further excluded because their preserved wound isolates did not regrow for biofilm analysis, while 54 were lost to follow-up. Finally, samples and data were analysed from 32 DFI patients. Figure 2A illustrates the patient recruitment procedure.

A total of eighteen HCs were screened for eligibility. Among them, two had taken vitamin D supplements in previous weeks. Consequently, 16 HCs met the eligibility criteria. Figure 2B illustrates the HC recruitment procedure.

### 4.3. Data Collection Methods

Most patient-related data, including demographics and clinical factors, were obtained from medical records. At four-month follow-up, the treating clinical team assessed the ulcer healing status and documented it in the patients’ medical records. Baseline ulcer dimensions were documented as length and width measurements. Ulcers recorded as “healed” and without pus or exudate were classified as healed, and those with residual dimensions were classified as unhealed ulcers. Healing was assessed using a binary clinical outcome (healed vs. unhealed) based on clinical records. Quantitative changes in wound area were not evaluated because calibrated wound photographs and systematic wound area measurements were unavailable.

### 4.4. Measurement of Serum Vitamin D, Vitamin D Receptor Protein, and LL-37 Levels in DFI Patients and HCs

Vitamin D, VDR protein, and LL-37 levels were measured in all 48 participants. A 4 mL blood sample was obtained from the participants and sent to KH for assessment of vitamin D levels using an electrochemiluminescence immunoassay on a COBAS 6000 analyzer (Roche Diagnostics GmbH, Mannheim, Germany). Another 4 mL blood sample was collected for ELISA to estimate serum VDR and LL-37 levels. Blood samples were centrifuged at 3000 rpm for 15 min to separate the serum, which was stored at −80 °C until analysis. VDR protein concentrations were measured using a Human VDR ELISA Kit (Elabscience, Houston, TX, USA; catalog number E-EL-H2043) according to the manufacturer’s instructions [40]. Similarly, LL-37 concentrations were determined using a Human Antibacterial Protein LL-37 ELISA Kit (Elabscience, USA; catalog number E-EL-H2438) [41]. For participants in the SG and RG, these assessments were repeated at 4-month follow-up to evaluate changes in serum vitamin D, VDR protein, and LL-37 levels. All patients and HCs provided informed consent.

### 4.5. VDR and CAMP Gene Expression Analysis by mRNA Quantitation in DFI Patients and HCs

VDR and CAMP gene expression analyses were conducted on all 48 participant samples from the HCs, RG, and SG [42]. Whole blood was used to extract total RNA using the Thermo Fisher Scientific PureLink RNA Extraction Kit (Waltham, MA, USA), following the manufacturer’s instructions. Before proceeding with downstream analysis, the extracted RNA was evaluated using a NanoDrop™ One Microvolume UV-Vis Spectrophotometer (Thermo Fisher Scientific, Inc., Waltham, MA, USA). The isolated RNA was then converted to complementary DNA (cDNA) using the Invitrogen SuperScript VILO cDNA synthesis kit (Invitrogen, Thermo Fisher Scientific, Waltham, MA, USA). The reverse transcription reaction was performed under the recommended thermal cycling conditions to produce high-quality cDNA suitable for quantitative analysis. Quantification of VDR and CAMP gene expression was performed using a cDNA template. TaqMan gene expression assays (Applied Biosystems, Thermo Fisher Scientific, Waltham, MA, USA) with assay IDs Hs01045843_m1 for VDR and Hs00189038_m1 for CAMP were performed according to the manufacturer’s protocol. Amplification and detection were performed using a QuantStudio™ 5 Real-Time PCR instrument (Applied Biosystems, Thermo Fisher Scientific, Waltham, MA, USA). Relative gene expression was calculated using the 2^−ΔΔCt^ method, with GAPDH (TaqMan gene expression assay ID: Hs03929097_g1) as the endogenous control. Blood samples were collected from the participants in the SG and RG after four months of follow-up. The analyses of VDR and CAMP gene expression were repeated using the same procedures. This four-month follow-up assessment was performed to evaluate changes in gene expression following physician-prescribed vitamin D supplementation.

### 4.6. Analysis of Biofilm Formation in Bacterial Isolates from Wound Samples of DFI Patients

Under sterile conditions, wound swabs or tissue samples were collected from the infected area for microbiological culture and biofilm analysis. Sixteen bacterial isolates obtained from wound samples of 11 patients in the SG were analyzed during a four-month follow-up for their biofilm-forming capabilities. Isolates were identified using the MALDI-TOF VITEK MS system (BioMérieux, Marcy l’Etoile, France), and antimicrobial susceptibility was determined using the VITEK 2 system (BioMérieux, Durham, NC, USA) [43].

Biofilm formation was measured using the tissue culture plate method [44,45]. Briefly, the isolates were grown in tryptone soy broth (TSB; HiMedia, Mumbai, India) supplemented with 1% glucose, diluted 1:100 in fresh medium, and incubated in 96-well polystyrene plates (Tarsons, Kolkata, India). *Staphylococcus aureus* (ATCC 25923) and *Pseudomonas aeruginosa* (ATCC 27853) were used as positive controls, and sterile TSB was used as the negative control. Following incubation, the wells were fixed with 2% sodium acetate, stained with 0.1% crystal violet, and quantified using a microplate reader (Multiskan Go; Thermo Fisher Scientific, Vantaa, Finland). All experiments were performed in triplicate. Interpretation of the biofilm formation by bacterial isolates was carried out in accordance with [46,47]. Results were interpreted as follows:

- ODei ≤ ODnc → non-biofilm producer;
- ODei > ODnc → biofilm producer [45].

where ODei is the optical density of each isolate, and ODnc is the optical density of the negative control.

### 4.7. Statistical Analysis

Jamovi 2.4.8.0 was used for data analysis. The Kruskal–Wallis test and post-hoc Dwass–Steel–Critchlow–Fligner (DSCF) test were used to compare baseline serum biomarker levels and relative VDR and CAMP gene expression among RG, SG, and HCs. Additionally, these tests were used to analyze four-month follow-up data from both the RG and SG and to compare them with the baseline data from the HCs. The association between vitamin D supplementation and ulcer healing, as well as the prevalence of biofilm-forming bacteria, was analyzed using Fisher’s exact test. Spearman’s correlation analysis was used to examine relationships among vitamin D, biomarkers, and the expression levels of the VDR and CAMP genes in the SG during a four-month follow-up.

## 5. Conclusions

Physician-prescribed vitamin D supplementation was associated with differences in serum vitamin D, VDR protein, LL-37, and peripheral blood VDR mRNA levels between the SG and RG. However, there was no significant difference in CAMP mRNA expression. As VDR and CAMP mRNA expression were analyzed in peripheral blood, the results may not be representative of the transcriptional activity in the wound microenvironment. Therefore, these findings should not be interpreted as direct evidence of local VDR–CAMP pathway activity. In summary, the present results provide preliminary evidence of a potential association between physician-prescribed vitamin D supplementation and the circulating elements of the vitamin D–VDR-CAMP/LL-37 pathway. However, future studies assessing both blood and wound tissue samples from the same patients are needed to clarify the relationship between systemic and local VDR–CAMP/LL-37 activities.

## Figures and Tables

**Figure 1:**
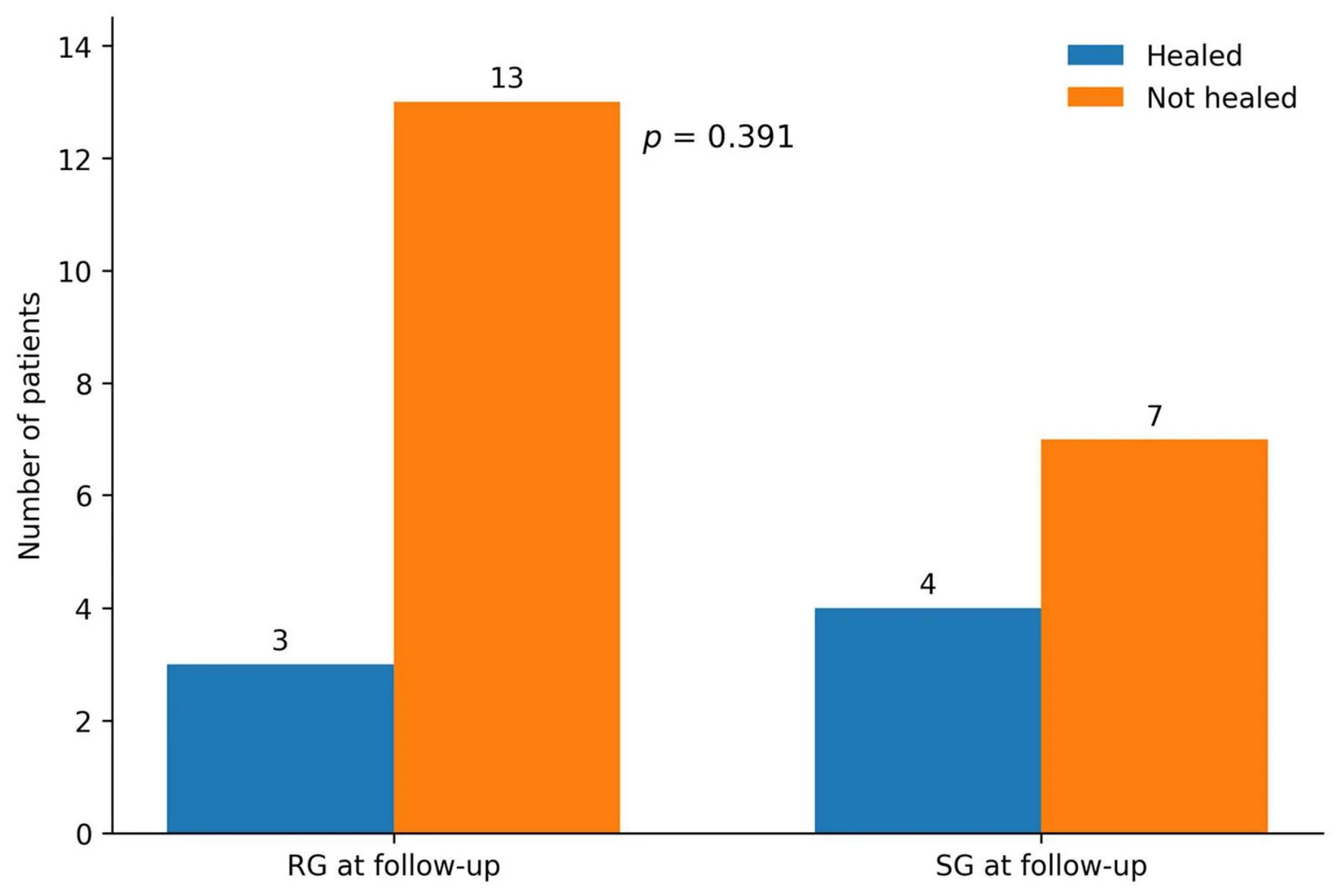
Comparison of wound outcome between RG and SG at four-month follow-up. RG: Reference group; SG: Study group.

**Figure 2:**
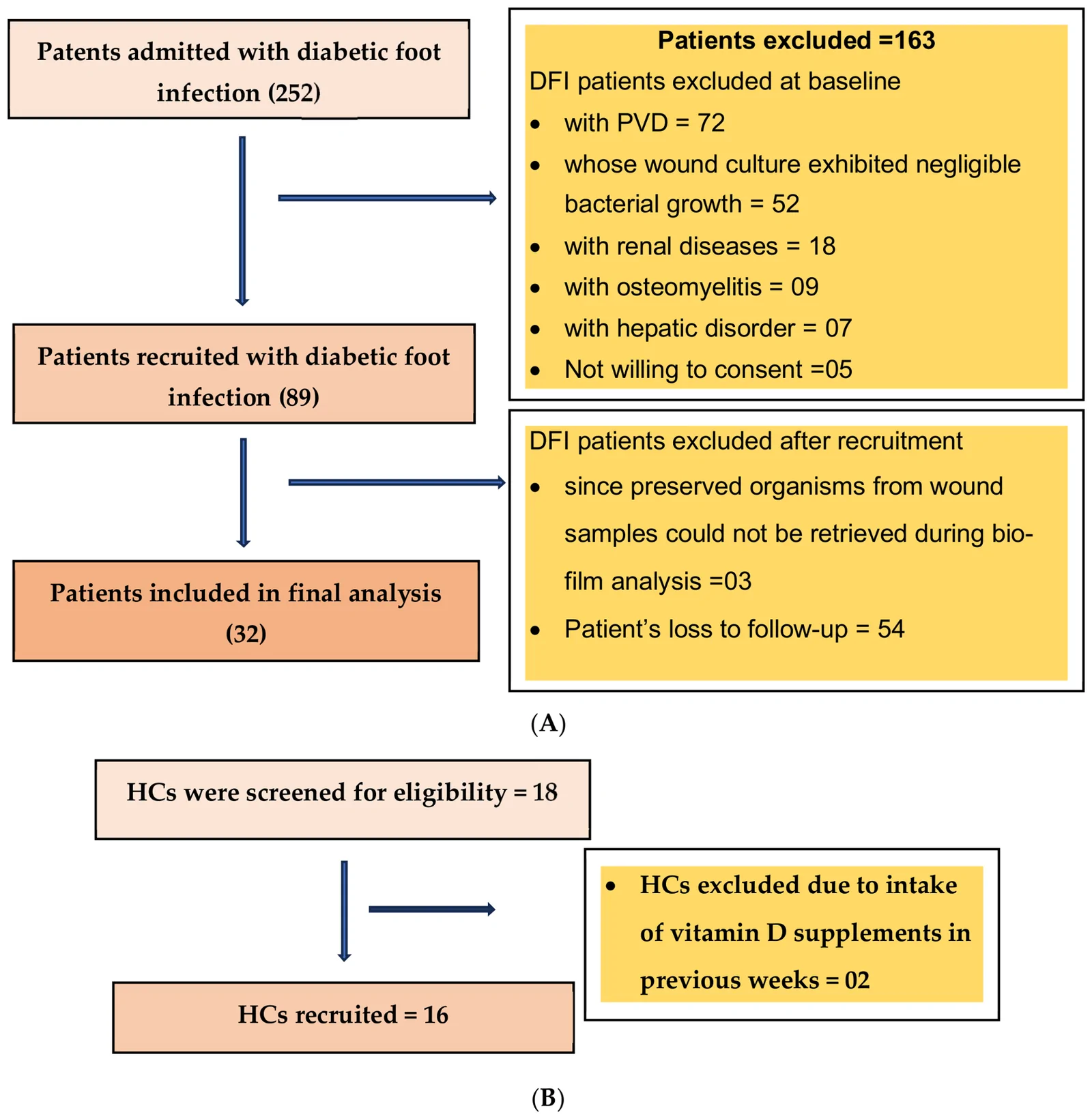
(**A**) Recruitment procedure of DFI patients. (**B**) Recruitment procedure of healthy controls.

**Table 1:** Comparison of characteristics of the study populations at baseline.

Variables ^#^	SG (*n* = 16)	RG (*n* = 16)	HCs (*n* = 16)	*p*-Value *
Age (years)	52.50 (10.00)	61.50 (7.50)	32.50 (9.25)	<0.001
Vitamin D (ng/mL)	11.40 (7.15)	16.30 (5.20)	21.90 (17.40)	0.001
VDR protein (ng/mL)	0.76 (0.34)	0.79 (0.41)	1.14 (0.39)	0.002
LL-37 (ng/mL)	1.03 (0.42)	1.26 (0.79)	1.89 (1.08)	0.001
VDR mRNA (fold change)	0.79 (0.64)	1.28 (1.34)	0.88 (2.47)	0.059
CAMP mRNA (fold change)	0.64 (0.52)	0.78 (0.54)	1.10 (1.91)	0.241

* Kruskal–Wallis test; ^#^ Data are expressed as median (IQR); SG: Study group; RG: Reference group; HCs: Healthy controls; VDR: Vitamin D receptor; LL-37: Cathelicidin; CAMP: Cathelicidin antimicrobial peptide; *n*: Sample size.

**Table 2:** Comparison of characteristics of the study populations at the four-month follow-up.

Parameter ^$^	SG (*n* = 11)	RG (*n* = 16)	HCs (*n* = 16)	*p*-Value *
Vitamin D (ng/mL)	21.3 ^a^	11.1 ^b^	21.90 ^a^	<0.001
VDR (ng/mL)	0.93 ^ab^	0.60 ^b^	1.14 ^a^	0.001
LL-37 (ng/mL)	1.89 ^a^	1.06 ^b^	1.89 ^a^	0.001
VDR mRNA (fold change)	1.31 ^a^	0.77 ^ab^	0.88 ^a^	0.067
CAMP mRNA (fold change)	1.10 ^a^	0.66 ^a^	1.33 ^a^	0.346

* Kruskal–Wallis test followed by Dwass–Steel–Critchlow–Fligner (DSCF); a, b different superscripts in rows indicate significant differences (*p* < 0.05) between groups. If the superscript a, or b is the same, there is no statistical difference between the groups; ^$^ Data are expressed as median; SG: Study group; RG: Reference group; HCs: Healthy controls; CAMP: Cathelicidin antimicrobial peptide; LL-37: Cathelicidin; *n*: Sample size; VDR: Vitamin D receptor; VDR and CAMP mRNA expression was calculated using the 2^−ΔΔCt^ method and is presented as non-dimensional relative fold change with respect to the HC group.

**Table 3:** Association of vitamin D status with biofilm-producing bacterial isolates in SG participants at four-month follow-up.

	Vitamin D Status			
Type of Bacteria	Deficient *n* (%)	Insufficient *n* (%)	Sufficient *n* (%)	*p*-Value *
Biofilm-forming bacterial isolates (*n* = 12)	8 (66.7)	3 (25.0)	1 (8.3)	0.163
Non-biofilm-forming bacterial isolates (*n* = 4)	1 (25.0)	1 (25.0)	2 (50.0)	

* Fisher’s exact test.

**Table 4:** Association of vitamin D status with ulcer healing outcomes in the SG participants at four-month follow-up.

Outcome	Vitamin D Status			*p*-Value *
	Deficient *n* (%)	Insufficient *n* (%)	Sufficient *n* (%)	
Healed (*n* = 4)	1 (25.0)	1 (25.0)	2 (50.0)	0.727
Unhealed (*n* = 7)	4 (57.1)	2 (28.6)	1 (14.3)	

* Fisher’s exact test.

**Table 5:** Correlation of vitamin D concentration with biomarkers and expression levels of the VDR and CAMP genes in SG patients at four-month follow-up.

Variables	Spearman Coefficient (r)	*p*-Value *
VDR protein	0.682	0.025
LL-37	0.825	0.002
VDR mRNA (fold change)	0.618	0.048
CAMP mRNA (fold change)	0.655	0.034

* Spearman rank correlation test; r: Spearman coefficient; VDR: Vitamin D receptor; LL-37: Cathelicidin; CAMP: Cathelicidin antimicrobial peptide; VDR and CAMP mRNA expression was calculated using the 2^−ΔΔCt^ method and is presented as non-dimensional relative fold change with respect to the HC group.

**Table 6:** Baseline characteristics of participants who completed the four-month follow-up and those lost to follow-up.

Variables ^#^	SG Participants		*p*-Value *
	Completed Follow-Up (*n* = 11)	Lost to Follow-Up (*n* = 5)	
Age (years)	56.00 (12.00)	68.00 (13.00)	0.11
Vitamin D (ng/mL)	14.50 (9.04)	10.60 (6.77)	0.58
VDR protein (ng/mL)	1.31 (0.77)	1.05 (0.84)	0.61
LL-37 (ng/mL)	0.79 (0.42)	0.57 (0.46)	0.60
VDR mRNA (fold change)	0.99 (0.35)	0.96 (0.22)	0.86
CAMP mRNA (fold change)	0.64 (0.52)	0.78 (0.54)	0.39

^#^ Data were expressed as median (IQR); * Mann–Whitney U test; *n* = sample size; VDR: Vitamin D receptor; LL-37: Cathelicidin; CAMP: Cathelicidin antimicrobial peptide; VDR and CAMP mRNA expression was calculated using the 2^−ΔΔCt^ method and is presented as non-dimensional relative fold change with respect to the HC group.

## Data Availability

The original contributions presented in this study are included in the article. Further inquiries can be directed to the corresponding author.

## Abbreviations

ATCC	American Type Culture Collection
CAMP	Cathelicidin antimicrobial peptide
DFI	Diabetic foot infection
DFU	Diabetic foot ulcer
DM	Diabetes Mellitus
DSCF	Dwass–Steel–Critchlow–Fligner
ECLIA	Electrochemiluminescence immunoassay
ELISA	Enzyme-linked Immunosorbent Assay
HbA1c	Glycated haemoglobin
HC	Healthy control
KH	Kasturba hospital
KMC	Kasturba Medical College
LL-37	Cathelicidin
mRNA	Messenger ribonucleic acid
n	Sample size
RG	Reference group
SG	Study group
TCP	Tissue culture plate
VDR	Vitamin D receptor
